# Silicone-Induced Granuloma of Breast Implant Capsule (SIGBIC): Histopathology and Radiological Correlation

**DOI:** 10.1155/2018/6784971

**Published:** 2018-09-20

**Authors:** Eduardo de Faria Castro Fleury, Gabriel Salum D'Alessandro, Sheila Cristina Lordelo Wludarski

**Affiliations:** ^1^Holy House School of Medicine, São Paulo, Brazil; ^2^Brazilian Cancer Control Institute, Brazil

## Abstract

Currently, attention has been given to complications related to breast implants, especially due to the presence of anaplastic large cell lymphoma (ALCL) related to silicone implants. Many manuscripts attempt to associate silicone presence with clinical complaints reported by patients, while others try to demonstrate the mechanisms of silicone bleeding by permeability loss of breast implant surfaces. There also are reports of foreign body type reactions from implant fibrous capsule to silicone corpuscles. However, there seems to be no study that correlates the clinical, radiological, and histological correlations of these lesions. The objective of this review is to correlate radiological findings of silicone-induced granuloma of breast implant capsule (SIGBIC) from breast MRI (BMRI) scans and complementary findings of ultrasound (US) and positron emission tomography (PET) scan, and its histology originated from surgical breast implant capsulectomy. To make this correlation possible, we divided SIGBIC into three radiological findings: (1) intracapsular SIGBIC, (2) SIGBIC with extracapsular extension, and (3) mixed SIGBIC associated with seroma. Our experience demonstrates histological-radiological correlation in SIGBIC diagnosis. Knowledge of these findings may demonstrate its real importance in terms of public health and patient management. We believe that SIGBIC is currently underdiagnosed by lack of training, guidance, and management in our clinical practice.

## 1. Introduction

Many complications are reported inherent to breast silicone implants, such as late seroma, infections, rejections, intra- and extracapsular ruptures, contractures, and more recently, anaplastic large cell lymphoma (ALCL) [1]. However, the determining factors to develop these complications are still a black box. Surprisingly, one of the most frequent BMRI findings at our service related to silicone implant complications is a granuloma induced by free silicone granules (SIGBIC), present in about 27.1% of cases.

This manuscript is based on data analysis obtained from an observational prospective study for breast implant evaluation in patients referred to a breast magnetic resonance scan. The study was approved by our Institutional Ethics Research Committee with an informed consent term signed by all patients. Patients with suggestive SIGBIC BMRI findings were recalled for additional ultrasonography and PET scan. Final diagnosis was confirmed by percutaneous biopsy or by surgical capsulectomy.

Since February 2017, 2891 BMRI have been analyzed. Of these, 830 patients were referred for breast implant evaluation. 27.1% of the implants presented BMRI signs of SIGBIC, of which 12.7% had associated intracapsular seroma and 3.3% had signs of extracapsular involvement.

At first, the study was designed to investigate the incidence of breast implant-associated anaplastic large cell lymphoma (BIA-ALCL). Since its beginning, we have not found any case of BIA-ALCL. However, we diagnosed a type of intracapsular lesion not reported in previous studies, where the imaging and clinical findings were very similar to BIA-ALCL. When correlating these findings to histological results, a granuloma induced by free silicone particles in fibrous capsule was found. Many of these cases were not diagnosed in the first histological report, where pathologists ignored to report free silicone presence. After the histological revision to investigate the presence of free silicone, SIGBIC was confirmed.

The lack of manuscripts in literature reporting SIGBIC imaging findings is especially due to the protocols adopted for BMRI breast implant evaluation, where contrast media is generally not used. This results in underdiagnoses of SIGBIC, especially with the differential diagnosis of late seroma [2].

More recent manuscripts still question whether BIA-ALCL would be a true lymphoma, assuming the possibility that it could be due to an immune response. In this context, a recent article was published which reported spontaneous regression of BIA-ALCL in 2 patients [3, 4].

The objective of this pictorial review is to illustrate how to describe the main imaging findings of silicone-induced granuloma of breast implant capsule (SIGBIC) and to correlate with cytopathology results.

## 2. SIGBIC Physiopathology

### 2.1. Breast Implant Surface Fatigue

It is speculated that any breast implant, whether saline or silicone, should bleed silicone corpuscles over time. Some factors can accelerate this, such as trauma determining microfractures, temperature or pressure exposure, ultraviolet radiation, oxidation, and chemical reactions.

**Figure 1:**
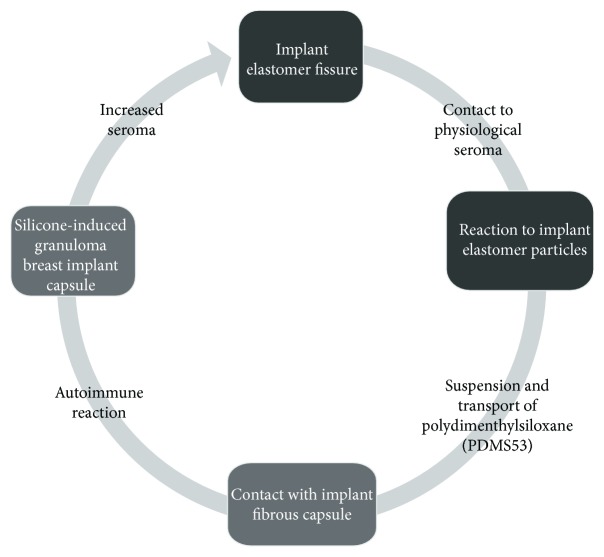
Schematic of silicone bleeding and transport of silicone components from the interior of the breast implant to the intracapsular region. Black boxes represent the environment of the breast implant. Gray boxes represent fibrous capsule. Gray arrow represents intracapsular space.

When permeability of elastomer is impaired, the physiological seroma that lies in the space between the elastomer surface and the fibrous capsule reacts and transports the resulting polydimethylsiloxane compound from the implant surface that will encounter the fibrous capsule [1, 5] (Figure 1).

### 2.2. Fibrous Capsule

Fibrous capsule consists of dense fibrosis, with an inner surface lined by pseudosynovia, consisting of a layer of histiocytes. When silicone particles get in contact with fibrous capsule, whatever the mechanism, an immune response could be generated from the host. Such response may be a silicone-induced granuloma, which may vary in intensity [6].

Fibrous capsule acts as physiological protection barrier against products from intracapsular environment. Blood supply to this intracapsular environment is restricted. This results in an indolent mass growth which predicts the good prognosis of SIGBIC when restricted to fibrous capsule. However, when fibrous capsule ruptures and exposes its contents to extracapsular space, the immune reaction becomes more exuberant. In these cases, silicone exposure can trigger a systemic immune reaction [6].

### 2.3. Immunological Response to Silicone

**Figure 2:**
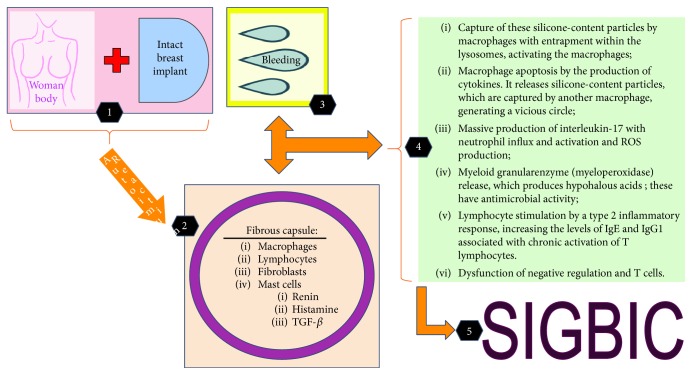
SIGBIC schematic formation resulting from the contact of the silicone granules with the fibrous capsule.

Some patients will develop an autoimmune reaction to silicone components, known as silicone implant incompatibility syndrome (SIIS). This immune response may range from indolent to more aggressive degrees [6, 7] (Figure 2).

**Figure 3:**
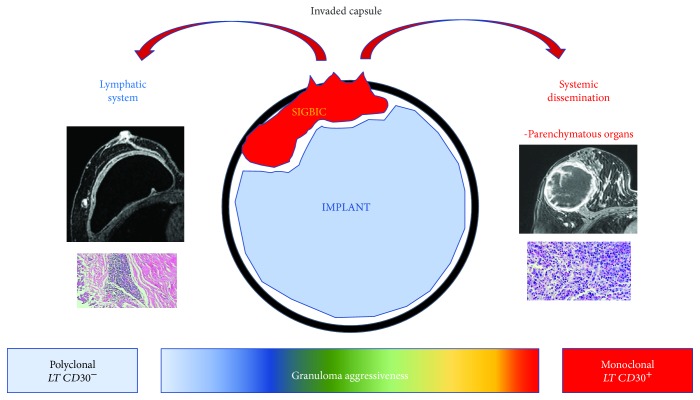
Hypothetic scheme of immune response aggressiveness to free silicone contact to fibrous capsule.

According to this immunological modulation, giant cells and lymphocyte relation will vary according to each host, from light when there is predominance of giant cells to more aggressive when there is a major lymphocyte component. At this point, great correlation between ALCL and SIGBIC is observed. However, ALCL has T lymphocyte CD30 positive to immunohistochemistry reaction [5] (Figure 3).

### 2.4. Silicone Particles

Silicone has several appearances at optical microscopy, maybe refractile but not polarized, appearing brighter and shinier than the normal tissue. If polarizable material is present, it is another type of material, not silicone. The two most frequent findings at histology are extracellular and intracellular silicones [7].

Extracellular silicone has clear spaces partially occupied by refractile (but not polarized) material amid giant cells, histiocytes, and lymphocyte appearances. On the other hand, intracellular silicone appears as light spheres that vary in size, located within histiocytes that sometimes looks like lipoblasts [7].

Histologically, SIGBIC is formed by extracellular and/or intracellular silicone, numerous histiocytes, chronic granulomatous inflammatory infiltrate with multinucleated giant cells, and infiltrate of mixed lymphocytes—T and B without atypia.

### 2.5. Immunohistochemistry

Immunohistochemistry profile may be used as an auxiliary tool to confirm the diagnosis of SIGBIC, especially to determine the differential diagnosis with ALCL. The diagnostic tests performed are

- CD3: T lymphocyte marker
- CD20: B lymphocyte marker
- CD30: marker of Hodgkin's disease, ALCL, and embryonal carcinoma
- CD68: marker of macrophages, monocytes, and Langerhans cells

As a rare differential diagnosis, ALCL reveals infiltrate of atypical lymphocytes with pleomorphic nucleus and 1 or more nucleoli. These lymphocytes may be arranged diffusely or in an aggregated distribution at the fibrous capsule. The mitotic index is usually higher, and necrosis may be present. Virtually, all these atypical cells have CD30 expression and negativity to anaplastic lymphoma kinase (ALK) [8].

## 3. Clinical Findings

Most SIGBICS are incident findings. The main clinical symptom is breast stiffening. Additional symptoms are skin rash, arthralgia, pruritus, and asthenia. These findings are similar to those described by de Faria Castro Fleury et al. where SIIS cases were compared over a 30-year period [9].

Patients usually present physical limitations to practice their daily activities, and some of them are submitted to specialized treatment with rheumatologists.

## 4. Histological-Radiological Correlation

For diagnostic and classification purposes, it was chosen to classify the SIGBIC according to BMRI findings. In this context, SIGBIC was divided into 3 categories:

1. Intracapsular SIGBIC
2. SIGBIC with extracapsular extension
3. Mixed SIGBIC associated with seroma

### 4.1. Intracapsular SIGBIC

These patients usually seek the breast specialist with a clinical complaint of capsular contracture, usually manifests as slow growth lesions. Symptoms may regress after use of anti-inflammatory and/or corticosteroid therapy. Definitive treatment is withdrawal of the causal factor, removing the implant and performing capsulectomy.

#### 4.1.1. Radiological Findings

Radiological SIGBIC findings were first reported by our group recently. To reach the definitive diagnosis, it is necessary for a trained radiologist to investigate the pathognomonic BMRI findings of SIGBIC. In addition to trained radiologists, it is imperative to use contrast medium and late dynamic sequences due to the difficulty of contrast reaching intracapsular environment [8].

When free silicone particles reach the implant fibrous capsule, a granuloma forms. This granuloma will present in BMRI as an intracapsular high signal in T2-weighted sequences. Most often, it exerts as external compressive effect on the implant surface. In these cases, there are no radiological signs suggestive of implant rupture. Usually, these lesions are erroneously classified as late seromas, especially by lack of use of contrast medium. SIGBIC can only be confirmed in the late dynamic sequences where late enhancement confirms the solid nature of the lesion [8, 10].

Another BMRI finding that allows the diagnosis is the black-drop sign. The black-drop sign corresponds to silicone granules within implant fibrous capsule, where marked low signal foci may be found. It is worth to mention that all cases of SIGBIC presented signs of capsular contracture.

**Figure 4:**
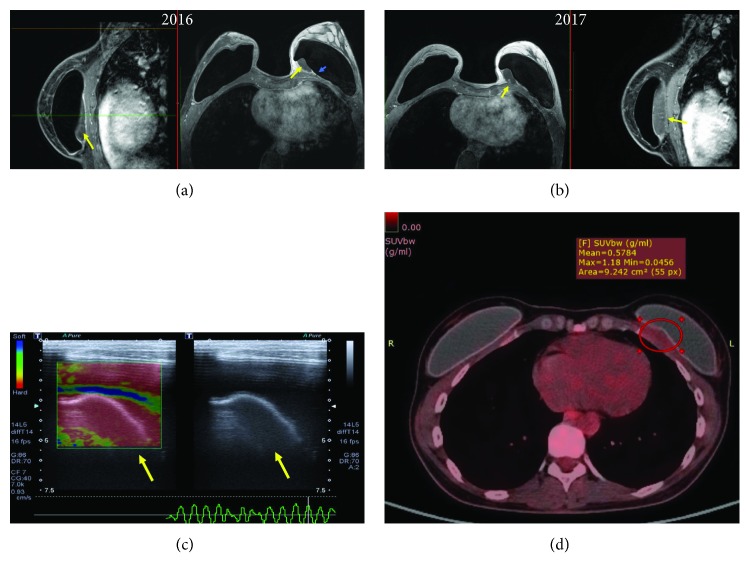
Intracapsular SIGBIC: imaging findings in a 40-year-old woman with left breast stiffening. (a) Breast MRI. The contrast BMRI shows an intracapsular mass with a slight contrast enhancement at coronal and axial images. Yellow arrows correspond to SIGBIC. Blue arrow corresponds to the black-drop signal. (b) One year after the first scan, the patient presented an increase of mass volume and of contrast enhancement. Yellow arrows correspond to SIGBIC. (c) Ultrasound. Intracapsular mass, posterior to the implant, with snowstorm-type aspect (yellow arrows). By elastography, it represents a hard lesion (left image). (d) PET scan. No significant radioisotope uptake by the mass (red circle) is observed. The radiological differential diagnosis is late seroma.

*(1) BMRI*. Breast MRI is the gold standard for diagnosis. The criteria for diagnosing SIGBIC described by our group are [8] (Figures 4(a) and 4(b))

- intracapsular mass with hypersignal at T2-weighted sequences
- black-drop signal in fibrous capsule
- late contrast enhancement at dynamic sequences, usually at after 4-minute phases

*(2) US*. Complementary ultrasound scan may aid the diagnosis of SIGBIC. The main finding is an intracapsular heterogeneous mass, which has the appearance of a snowstorm type, related to free silicone. Doppler scan will generally not present vascularization, and elastography is hard (Figure 4(c)).

*(3) PET Scan*. PET scan can assist in determining whether there is involvement of the extracapsular compartment. When intracapsular, PET scan scanning tends to be negative (Figure 4(d)).

#### 4.1.2. Histological Findings

**Figure 5:**
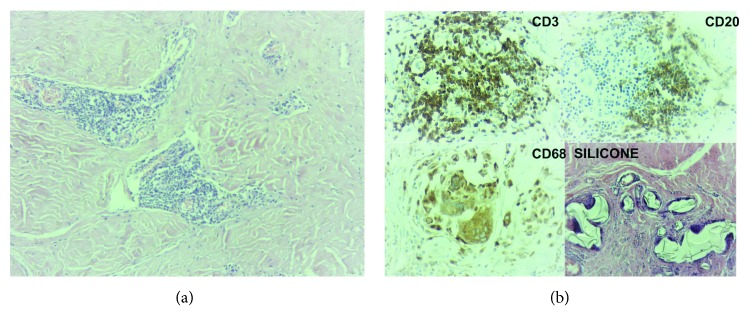
Intracapsular SIGBIC microscopy and immunochemistry of the same patient of Figure 4. (a) Optical microscopy. Small foci of aggregate lymphocyte associated with a moderate chronic inflammatory process. The left image in smaller augmentation and the right one in greater augmentation. (b) Immunohistochemistry. Positivity for CD3, CD20, and CD68 reactions. There is also a predominance of extracellular silicone (last image).

Predominance of extracapsular silicone with a lower aggregate lymphocyte population is observed, usually found in fibrous capsule focal thicket areas. It can be associated with a moderate chronic foreign body inflammatory process type and rare xanthomatous histiocytes. No individualized lymphocytes dispersed by the fibrous capsule or atypical cells are observed (Figures 5(a) and 5(b)).

### 4.2. SIGBIC with Extracapsular Extension

SIGBIC with extracapsular extension is related to fibrous capsule invasion. It usually has edema of the breast tissue at the periphery of the prosthesis. The symptomatology is more exuberant, where a recent history of volumetric breast enlargement, accompanied by stiffness and local phlogistic signs, are often reported.

#### 4.2.1. Radiological Findings

*(1) BMRI*. In addition to the solid component reported, small seroma may be present. Capsular invasion is demonstrated by involvement of the breast tissue at the fibrous capsule periphery. Lymph nodes with siliconomas can also be visualized.

**Figure 6:**
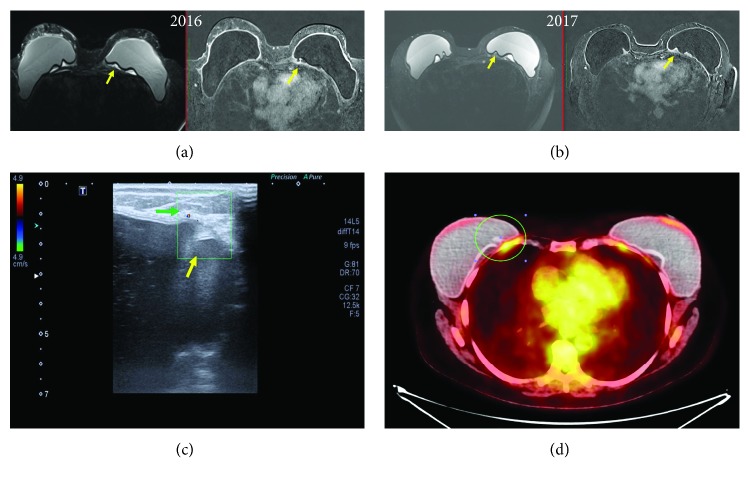
SIGBIC with extracapsular extension. 52-year-old woman with left breast stiffening. (a) T2-weighted BMRI shows an intracapsular hypersignal mass posterior to the implant (left image). After contrast, there is small enhancement of the mass (right image). (b) One year after the first scan, the patient presented an increase of mass volume and of contrast enhancement. Yellow arrows correspond to SIGBIC. Diffuse thickening of the fibrous capsule is also observed. (c) Ultrasound. Doppler scan shows an increase in vascularization at the periphery of the implant, where an intracapsular mass is observed, determining a snowstorm-type appearance. There are also changes of the inner implant echotexture. (d) PET scan. Radioisotope uptake by the mass (green circle). There is also uptake at the ticked fibrous capsule. The radiological differential diagnosis are late seroma, infectious disease, and BIA-ALCL.

As there is loss of fibrous capsule barrier and host response to the foreign body, earlier contrast enhancement of the involved areas is observed (Figures 6(a) and 6(b)).

*(2) US*. Most often, it is possible to find inner texture changes of the breast implants, with areas of a snowstorm type at fibrous capsule. Peripheral increased vascularization may be observed (Figure 6(c)).

*(3) PET Scan*. With the breakage of the fibrous capsule protection, uptake of radioisotope by granuloma is seen. Lymph nodes in the axillary chains and/or in distant chains may be found (Figure 6(d)).

#### 4.2.2. Histological Findings

**Figure 7:**
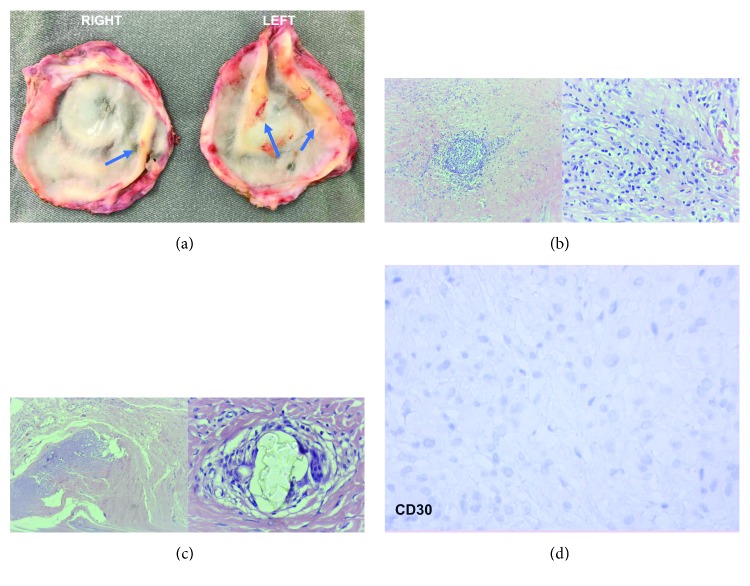
Pathology SIGBIC with extracapsular extension: imaging findings of the same patient of Figure 6. (a) Macroscopy of the fibrous capsule. Firm-elastic masses are observed in the intracapsular face (blue arrows). (b) Microscopy. The germinal center of B and T lymphocytes is observed by optical microscopy (smaller increase to the right and larger increase to the left). It is distinguished from intracapsular SIGBIC especially by the presence of lymphocytes individualized diffusely by fibrous capsule, predominantly T lymphocyte. (c) Microscopy. Right figure shows fibro necrotic material while left stands out the extracellular silicone. (d) Immunohistochemistry. There is no CD30 reaction.

At macroscopy, SIGBIC will appear as masses attached to the fibrous capsule inner surface, with firm-elastic consistence and yellow-whitish color. Another indication of its presence is breast implant color change, from white to yellow, known as a cloudy implant (Figure 7(a)).

SIGBIC with extracapsular extension has association of intracellular and extracellular silicones, with predominance of the extracellular type. Generally, there is a moderate chronic inflammatory process, foreign body-like reaction, and moderate xanthomatous histiocyte amount.

It is distinguished from the intracapsular SIGBIC by the presence of multiple aggregates of mature and nonatypical lymphocytes (immunophenotypes B and T), some of which have germinal centers. There is still a dense diffuse lymphocyte infiltration without atypia by the capsule.

Associated findings, capsular calcifications, and the presence of fibro necrotic material may be found (Figures 7(b)–7(d)).

### 4.3. Mixed SIGBIC Associated with Seroma

Symptomatology is quite exuberant in these cases, where abrupt volumetric increase of the affected breast accompanied by phlogistic signs is reported.

#### 4.3.1. Radiological Findings

**Figure 8:**
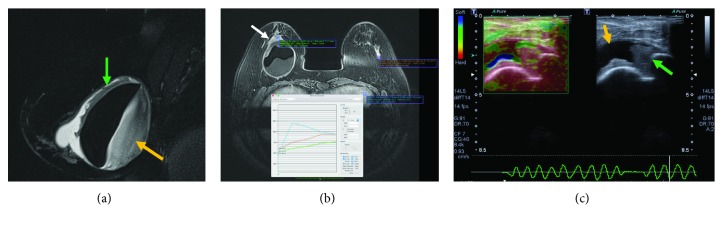
Mixed SIGBIC with seroma: imaging findings. (a) BMRI. Sagittal T2-weighted sequence presenting a large seroma with thick seroma content (yellow arrow). There are also enhanced masses on the anterior surface (green arrow). (b) Dynamic postcontrast sequence. Extracapsular mass (white arrow) that presents the type I kinetic curve pattern (persistent—green curve), inferring vascularized lesion. (c) Ultrasonography with elastography. Orange arrow points to the seroma component and the green arrow to the solid component. The seroma component is soft by elastography while the solid is hard. The radiological differential diagnosis are infectious disease and BIA-ALCL.

*(1) BMRI*. In addition to the findings described in SIGBIC with extracapsular extension, large seroma formation is observed. Seroma must have a thick serous content, with a heterogeneous signal in the T2-weighted sequences. After the injection of the contrast medium, there may be enhancement of coarse septa which communicate with the fibrous capsule (Figures 8(a) and 8(b)).

*(2) US*. Seroma is easy to identify with ultrasound and is often associated with debris. The mass content can also be visualized and usually has increased vascularization at Doppler scans (Figures 8(c)).

#### 4.3.2. Histological Findings

**Figure 9:**
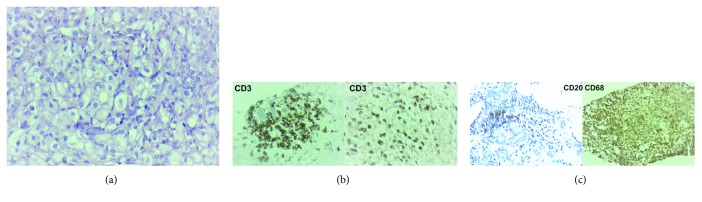
Histology of mixed SIGBIC associated with seroma of the same patient of Figure 8. (a) Optical microscopy. Prevalence of intracellular silicone, with light spheres that vary in size, located within histiocytes. (b) Immunohistochemistry. CD3 reaction. Prevalence of T lymphocyte, both in the germinal center (right image) and dispersed by the capsule (left image). (c) Immunohistochemistry. CD20 reaction shows B lymphocyte reaction (left figure), and CD68 shows histiocyte reaction (left image).

Despite seroma formation, histology of mass content is very similar to the extracapsular extension type, with intra- and extracellular silicones. However, in these cases, the intracellular silicone predominates (Figures 9(a)–9(c)).

**Figure 10:**
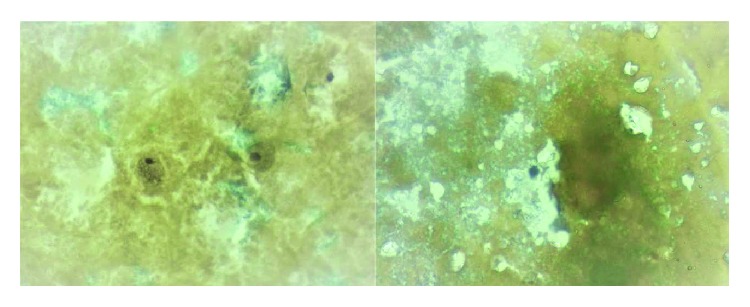
Mixed SIGBIC with seroma cytology of the same patient of Figures 8 and 9. Left image shows rare degenerated histiocytes while the right rare lymphocytes.

Seroma cytology may demonstrate degenerate histiocytes and rare small lymphocytes without atypia (Figures 10).

## 5. Conclusion

Our experience demonstrates histological-radiological correlation to SIGBIC diagnosis (Table 1). Knowledge of these findings by radiologists and pathologists improve its diagnosis. We believe that SIGBIC is currently underdiagnosed by lack of training, guidance, and management at our clinical practice.

## Figures and Tables

**Table 1:** Main keys for the diagnosis of silicone-induced granuloma of breast implant capsule (SIGBIC) by breast magnetic resonance imaging (BMRI) and histology.

SIGBIC	BMRI	Histology
(1) Intracapsular	(i) Restricted to intracapsular space (ii) Mass with hypersignal at T2-weighted sequences (iii) Black-drop signal (iv) Late contrast enhancement	(i) Extracellular silicone (ii) Lower population of aggregate lymphocytes (iii) Moderate chronic inflammatory process

(2) Extracapsular extension	(i) Same findings from intracapsular (ii) Pericapsular edema (iii) Lymph node involvement (iv) Early contrast enhancement of the extracapsular content	(i) Extracellular and intracellular silicone (ii) Predominance of extracellular silicone (iii) Germinal centers of lymphocytes (iv) Diffuse infiltration by lymphocytes at the fibrous capsule

(3) Mixed with seroma	(i) Same findings from the others (ii) Voluminous seroma	(i) Extracellular and intracellular silicone (ii) Predominance of intracellular silicone (iii) More severe infiltration by lymphocytes at the fibrous capsule (iv) Seroma of histiocytes and lymphocytes

## Abbreviations

SIGBIC: Silicone-induced granuloma of breast implant capsule
ALCL: Anaplastic large cell lymphoma
BMRI: Breast magnetic resonance imaging
US: Ultrasound
PET: Positron emission tomography
PDMS: Polydimethylsiloxane
SIIS: Silicone implant incompatibility syndrome
ALK: Anaplastic lymphoma kinase.
